# Avacopan in Anti-Neutrophil Cytoplasmic Autoantibodies–Associated Vasculitis in a Real-World Setting

**DOI:** 10.1016/j.ekir.2024.07.007

**Published:** 2024-07-06

**Authors:** Jonas Zimmermann, Janis Sonnemann, Wolfram J. Jabs, Ulf Schönermarck, Volker Vielhauer, Markus Bieringer, Udo Schneider, Ralph Kettritz, Adrian Schreiber

**Affiliations:** 1 Department of Nephrology and Medical Intensive Care, Charité - Universitätsmedizin Berlin, Germany; 2 Experimental and Clinical Research Center, Charité - Universitätsmedizin Berlin and Max Delbrück Center for Molecular Medicine in the Helmholtz Association, Berlin, Germany; 3 Department of Nephrology, Vivantes Klinikum im Friedrichshain, Berlin, Germany; 4 Department of Medicine IV, Division of Nephrology, LMU University Hospital, LMU Munich, Germany; 5 Department of Cardiology and Nephrology, Helios Klinikum Berlin -Buch, Germany; 6 Department of Rheumatology and Clinical Immunology, Charité - Universitätsmedizin Berlin, Germany

**Keywords:** anti-neutrophil cytoplasmic autoantibodies (ANCA), avacopan, complement C5a, necrotizing crescentic glomerulonephritis, vasculitis

## Introduction

Anti-neutrophil cytoplasmic antibody (ANCA)–associated vasculitis (AAV) represents a group of systemic autoimmune small vessel vasculitis, leading to significant morbidity and mortality due to extensive organ damage (including kidney failure)^1^ and treatment toxicity to medications such as glucocorticoids.^2^ Thus, research focuses on developing treatment that minimize glucocorticoid use.

AAV pathogenesis involves the activation of the alternative complement pathway, producing C5a.^3^ Avacopan, by targeting the C5a receptor CD88, inhibits neutrophil activation.^3^^,^^4^ In recent studies, treatment with avacopan reduced steroid exposure and adverse events (AEs) while increasing sustained remission and improving kidney function, leading to its approval in Germany in January 2022.^5^, ^6^, ^7^ However, limited real-world experience with avacopan beyond clinical trials underscores the importance of further evaluations, particularly for patients with estimated glomerular filtration rate (eGFR) of <15 ml/min and those with diffuse alveolar hemorrhage (DAH) with mechanical ventilation, who have not yet been studied. We performed a multicenter observational study in patients with AAV to explore treatment response, glucocorticoid use, tolerability, safety, and the rationale to opt for avacopan in a real-world clinical setting.

## Results

A total of 39 patients were included in the analysis with a mean follow-up period of 41 weeks (range, 12–56 weeks) and a mean Birmingham Vasculitis Assessment Score (BVAS) of 17 points (Table 1). Patient selection and end points are described in the Supplementary Methods. Renal involvement was observed in 85% of cases. At the time of diagnosis, 15 patients (38%) presented with an eGFR < 15 ml/min with 7 patients (18%) requiring dialysis. Furthermore, DAH occurred in 7 patients (18%) with 2 necessitating invasive ventilation. Additional patient characteristics are depicted in Table 1.

**Table 1:** Patient and clinical characteristics, treatment, and primary and secondary end points

Characteristic					
Sex, *n* (%)					
Female		18/39 (46)			
Male		21/39 (54)			
Age, yr (mean; IQR)		64 (51–72)			
Diagnosis, *n* (%)					
PR3-ANCA		22/39 (56)			
MPO-ANCA		15/39 (38)			
MPO-ANCA + anti-GBM		2/39 (5)			
Newly diagnosed/relapse, *n* (%)					
Newly diagnosed		20/39 (51)			
Relapsed		19/39 (49)			
BVAS (at time of diagnosis), mean		17			
Organ involvement, *n* (%)					
General		24/39 (62)			
Renal		33/39 (85)			
eGFR <15 ml/min		15/39 (38)			
Initially dialysis-dependent^a^		7/39 (18)			
Pulmonal		20/39 (51)			
Ear, nose, and throat (ENT)		12/39 (31)			
Cutaneous		5/39 (13)			
Mucous membranes or eyes		6/39 (15)			
Cardiovascular		3/39 (8)			
Nervous system		7/39 (18)			
Abdominal		1/39 (3)			
Treatment					
Induction therapy, *n* (%)					
Methylprednisolone pulse		30/39 (77)			
Rituximab (initially)^b^		24/39 (62)			
Cyclophosphamide (initially)^c^		15/39 (38)			
Plasma exchange		11/39 (28)			
Maintenance therapy, *n* (%)					
Avacopan		39/39 (100)			
Prednisolone		37/39 (95)			
Rituximab^d^		21/27 (78)			
Mycophenolate mofetil		3/27 (11)			
Azathioprine		2/27 (7)			
Methotrexate		1/27 (4)			
Primary endpoints					
Remission at 6 mo^e^, *n* (%)		28/32 (87.5)			
Sustained remission at 12 mo^f^, *n* (%)		21/23 (91)			
Secondary end points					
Subgroup eGFR < 15 ml/min					
Remission at 6 mo^e^, *n* (%)		11/12 (92)			
Sustained remission at 12 mo^f^, *n* (%)		8/9 (89)			
Subgroup DAH					
Remission at 6 mo^e^, *n* (%)		5/6 (83)			
Sustained remission at 12 mo^f^, *n* (%)		3/3 (100)			
Relapse^g^		4/39 (10)			
Secondary end points (continuation)	At diagnosis	mo 1	mo 3	mo 6	mo 12
	*n* = 39^h^	*n* = 39^h^	*n* = 39^h^	*n* = 32^h^	*n* = 23^h^
BVAS (version 3)	17 (7)	6 (3)	4 (4)	0 (1)	0 (0)
Renal BVAS	10 (4)	4 (2)	3 (3)	0 (0)	0 (0)
CRP (mg/l)	52.7 (59.6)	8.3 (14.8)	7.2 (25.6)	4.0 (11.0)	3.9 (9.2)
MPO/PR3-ANCA titer (U/ml)	131.8 (68.9)	100.9 (68.2)	71.2 (63.9)	58.0 (63.1)	42.1 (39.0)
Serum-creatinine (mg/dl)	3.69 (4.65)	2.20 (1.51)	1.79 (1.08)	1.73 (1.13)	1.64 (1.21)
eGFR (ml/min)	37 (36)	41 (32)	44 (29)	44 (29)	51 (31)
UACR (g/g creatinine)	0.99 (1.10)	0.90 (1.07)	0.59 (0.78)	0.35 (0.49)	0.17 (0.19)
UPCR (g/g creatinine)	1.46 (1.56)	1.41 (1.57)	0.83 (1.01)	0.49 (0.55)	0.45 (0.76)
Hematuria, *n* (%)	34/39 (87)	26/35 (74)	21/36 (58)	13/31 (42)	3/21 (14)
Subgroup eGFR < 15 ml/min	At diagnosis	mo 1	mo 3	mo 6	mo 12
	*n* = 15^h^	*n* = 15^h^	*n* = 15^h^	*n* = 12^h^	*n* = 9^h^
eGFR (ml/min)	8 (8)	22 (16)	30 (25)	33 (31)	35 (38)

BVAS, Birmingham Vasculitis Activity Score; CRP, C-reactive protein; DAH, diffuse alveolar hemorrhage; eGFR, estimated glomerular filtration rate, GBM, glomerular basement membrane; GPA, granulomatosis with polyangiitis; MPO, myeloperoxidase; MPA, microscopic polyangiitis; PR3, proteinase 3; UACR, urine albumin-to-creatinine ratio; UPCR, urine protein-to-creatinine ratio.

a The average duration of dialysis was relatively short in 6 of those patients (11 days). In addition, 1 patient required permanent dialysis.

b Eight Patients, who initially received cyclophosphamide were switched to rituximab therapy.

c Two Patients, who initially received rituximab were switched to cyclophosphamide therapy.

d In 5 additional patients, maintenance therapy with rituximab was planned.

e BVAS of 0 and ≤7.5mg prednisolone at 6 months and no relapse within the first 6 months.

f BVAS of 0 and ≤7.5mg prednisolone at 6 and 12 months and no relapse.

g Major relapse with intensification of immunosuppressive therapy. Organ involvement: 1× renal, 1× cardial, 1× pulmonary, 1× neurological and ENT; time of relapse: 4.7 months (mean), range: 3 to 9 months; treatment (initial): methylprednisolone (3/4 patients), rituximab (1/4 patients), cyclophosphamide (3/4 patients), prednisolone taper (4/4 patients), avacopan (4/4 patients); maintenance treatment: 1× azathioprine which was changed to 5m prednisolone due to GI intolerance (other patients still without maintenance therapy); treatment after relapse: plasmapheresis (1/4 patients), cyclophosphamide (1/4 patients, prior rituximab), rituximab (3/4 patients, prior cyclophosphamide), prednisolone taper (3/4 patients), continuation of avacopan (4/4 patients).

h Mean (± SD), unless stated otherwise.

Induction therapy was initiated with rituximab in 62% of cases (24/39) and cyclophosphamide in 38% (15/39). Plasma exchange was received by 28% of patients (11/39). All patients received a remission maintenance treatment, with rituximab being the most common (78%; 21/27). A summary of therapy is presented in Table 1.

**Figure 1:**
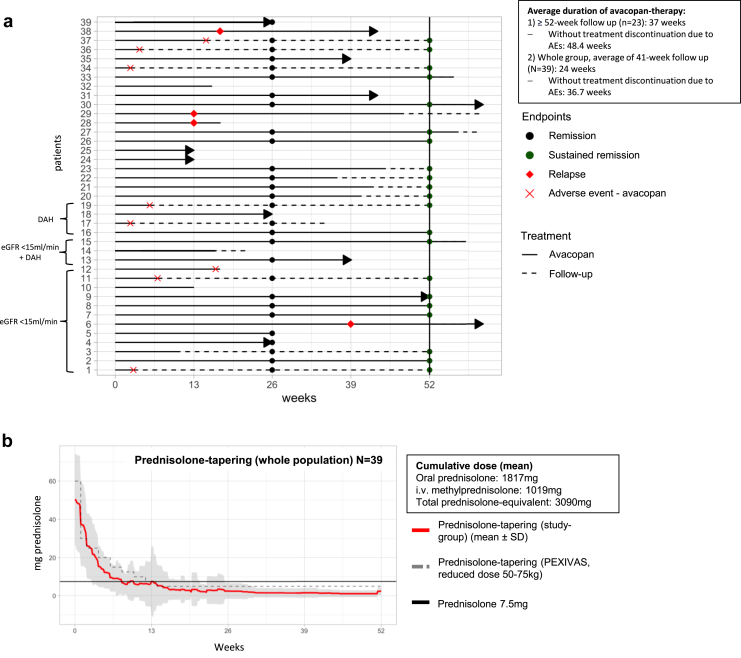
(a) Course of individual patients (primary end points, duration of avacopan treatment) and prednisolone tapering during 12-month follow-up. All included patients are shown over time (weeks) and sorted by subgroups (eGFR < 15 ml/min, DAH). Three patients had both eGFR < 15 ml/min and DAH and were assigned to both groups. The black dots at week 26 indicate remission (28/32 patients), and the green dots at week 52 represent sustained remission (21/23 patients). If there are no dots at these time points, remission or sustained remission was not reached. Reasons for not achieving remission include 2 relapses, 1 BVAS > 0, and 1 prednisolone dose > 7.5 mg/d; reasons for not achieving sustained remission include 2 relapses. The black lines represent the duration of avacopan therapy, with arrows indicating ongoing use. Due to the retrospective nature of the analysis, the exact timing of avacopan initiation is unknown and is therefore represented as day 0 in the figure. Red crosses at the end of a black line indicate that an adverse event (AE) caused therapy discontinuation. The dashed line represents the follow-up period. The average duration of avacopan therapy among patients with a ≥52-week follow-up and for the whole group is shown in the black box. Therapy with avacopan was discontinued in 9 patients (23%), of which 8 cases (21%) were due to AEs: fever and leukopenia (2 patients), significant increases in transaminase levels (2 patients), intolerable gastrointestinal side effects (2 patients), severe cough, and respiratory mucus production (2 patients). (b) The average daily oral prednisolone dose (mean ± SD, represented by the red solid line) is shown for the entire study population (*n* = 39). For comparison, the prednisolone tapering regimen from the PEXIVAS study (reduced dose for 50–75 kg of body weight, mean, depicted by the dashed line) is illustrated. The black line indicates a prednisolone dose of ≤7.5 mg. In addition, the oral prednisolone dose, i.v. methylprednisolone dose, and the total prednisolone equivalent dose are provided. AEs, adverse event; BVAS, Birmingham Vasculitis Activity Score; DAH, diffuse alveolar hemorrhage; eGFR, estimated glomerular filtration rate, PEXIVAS, plasma exchange and glucocorticoids in severe antineutrophil cytoplasmic antibody-associated vasculitis.

Remission was achieved in 87.5% (28/32) at 6 months and sustained remission in 91% (21/23) at 12 months. The remission rates of both subgroups (eGFR < 15 ml/min and DAH) were comparable to those of the overall cohort. However, it is important to note the small sample size, particularly among the patients with DAH (Table 1). An overview of the course of individual patients (remission, sustained remission, and follow-up time) is provided in Figure 1a. In the overall cohort and both subgroups, the BVAS score, ANCA titer, and C-reactive protein levels declined rapidly. The mean eGFR (entire cohort) increased from 37 to 51 ml/min per 1.73 m^2^ over 12 months. Notably, patients with an initial eGFR < 15 ml/min showed an increase from 8 to 35 ml/min (Table 1) and patients with DAH showed an increase from 40 to 66 ml/min. Albuminuria nearly completely resolved after 12 months. However, it initially decreased only gradually in the overall cohort and among patients with DAH. In patients with eGFR < 15 ml/min, there was a slight increase in the first month before it began to decline (Supplementary Figure S1). Four patients (10%) experienced a major relapse. A summary of relapse timing, organ involvement, and treatment is provided in Table 1. Primary and secondary end points are summarized in Table 1 and Supplementary Figure S1A–R; parameters for each patient are shown in Supplementary Figure S2 and S3A–H.

The mean prednisolone equivalent dose per patient was 3090 mg (*n* = 39) (Figure 1b). Excluding relapses, the mean dose was 2688 mg (*n* = 35) (Supplementary Figure S4). Among patients with a follow-up of ≥52 weeks (*n* = 23), the average duration of avacopan therapy was 37 weeks, extending to 48 weeks when excluding those with discontinuation due to AEs (Figure 1a). Avacopan was discontinued before the planned course in 9 patients (23%), of which, 8 cases (21%) were due to AEs, including fever or leukopenia (*n* = 2), increased transaminase levels (*n* = 2), intolerable gastrointestinal effects (*n* = 2), and cough with respiratory mucus, significantly affecting quality of life (*n* = 2) (Figure 1a). The discontinuation rate was 20% (3/15) for patients with eGFR < 15 ml/min and 29% (2/7) for those with DAH. Upon discontinuation of avacopan, symptoms and laboratory parameters improved in all affected patients.

Of the 39 patients, 14 (36%) experienced at least 1 serious AE, with 3 cases (8%) classified as life-threatening. One patient died of an unknown cause after ceasing to attend cyclophosphamide therapy sessions. Infections occurred in 14 patients (36%), with 6 (15%) classified as “serious.” Potential glucocorticoid-associated AEs (Supplementary Table S1) were observed in 24 patients (62%), mainly involving cardiovascular, infectious, and endocrine or metabolic issues. Patients with eGFR < 15 ml/min or DAH exhibited comparable rates of AEs and serious AEs. However, infection rates and serious infections were higher in patients with initial eGFR < 15 ml/min compared to the overall cohort (36% vs. 60% and 15% vs. 27%, respectively) (Supplementary Table S2).

The primary reason for prescribing avacopan was the intention to reduce the cumulative glucocorticoid dose (77%), followed by enhancing renal response (59%), improving overall therapeutic outcomes (54%), and intensifying treatment for uncontrolled disease or desire for higher immunosuppressive efficacy or relapse (51%) (Supplementary Table S3).

## Discussion

This multicentric observational study assessed avacopan efficacy and safety, as well as the decision-making factors in a real-world setting. Notably, the initial BVAS of 17 in our study closely aligns with the ADCOVATE trials’ severity (BVAS of 16); however, we observed higher rates of remission at 6 months (87.5%) and sustained remission at 12 months (91%), exceeding the 72.3% and 65.7% in the ADCOVATE trial.^5^ Remarkably, we included patients with severe conditions such as eGFR < 15 ml/min and DAH with some requiring invasive ventilation, who were excluded in previous studies. Nevertheless, both subgroups also demonstrated high remission rates, which had not been previously investigated. However, the small sample size, especially among patients with DAH, warrants cautious interpretation. Further prospective studies in these subgroups are needed to validate these findings. It is essential to note that the definition of remission varied between studies, which could contribute to higher remission rates: patients were considered in remission even with a prednisolone dose of ≤7.5 mg, whereas in the ADVOCATE trial, patients had to be off glucocorticoids for 4 weeks before end point assessment at 26 and 52 weeks. We chose this definition because, in clinical practice, patients sometimes maintain a low dose of glucocorticoids while still being clinically in remission. Furthermore, our patients had a more intensive immunosuppression; as at month 6, 11 of 32 patients were still taking prednisolone (1.25–5 mg; 1 patient taking 10 mg) and at month 12, 8 of 23 patients were still taking prednisolone (1.25–5 mg). The prolonged prednisolone use in patients in remission reflects real-world management rather than disease activity. Conceivably, the initiation of a remission maintenance therapy for all patients, mostly with rituximab, likely improved sustained remission rates while maintaining a low relapse rate (10%). No increase in relapse rates was observed in the subgroups. Minor relapses were not detected in this study.

Despite the severely affected patient cohort, our study showed substantial improvements in eGFR, particularly in patients with an initial eGFR < 15 ml/min, which had not been demonstrated before. Our study confirmed the positive impact on (kidney-related) outcomes, including improvements in hematuria, albuminuria, BVAS scores, C-reactive protein levels, and ANCA titers. These improvements were also observed in both subgroups. However, the rapid decline in albuminuria seen in the ADVOCATE trial was not replicated. In patients with eGFR < 15 ml/min, there was even a slight increase in albuminuria in the first month, followed by a decline until nearly complete resolution after 12 months. This slower decline could be due to more severe kidney involvement and the retrospective nature of the analysis, which left the exact timing of avacopan initiation unknown, potentially indicating it was not started at AAV onset. In addition, the lack of a control group precludes direct comparison of results without avacopan.

In our study, the cumulative prednisolone dose at 52 weeks was 3090 mg, surpassing the ADVOCATE study's total of 2002.9 mg, but was significantly lower than in PEXIVAS (reduced dose arm).^8^ We believe physicians did not fully utilize the steroid-sparing effect of avacopan. This may be due to a lack of awareness among clinicians and the absence of regular, standardized control visits in clinical practice compared to randomized controlled trials, leading to higher steroid dosages.

Whereas the ADVOCATE study,^5^ mandated a 52-week treatment, our study observed an average avacopan use of 37 weeks, extending to 48 weeks when excluding therapy discontinuations, close to the pivotal study. Specific criteria for avacopan use in AAV treatment are not clearly defined.^9^ In our study, the main reason for its use was to reduce glucocorticoids and enhance patient and renal outcomes. In addition, avacopan was employed for severe, relapsing, or refractory cases, achieving high remission rates. Therefore, future studies should focus on prospectively evaluating avacopan efficacy in patients with relapses or resistance to conventional treatments.

Our study focused on the safety and tolerability of avacopan, which was discontinued due to AEs in 21% of cases, with no serious AEs reported. This rate closely aligns with the ADVOCATE trial (15.7%).^5^ Patients with eGFR < 15 ml/min had a discontinuation rate of 20%, whereas those with DAH had a rate of 29%, with the caveat of a small sample size. Following discontinuation, clinical and laboratory improvements were noted without permanent damage. The incidence of AEs, serious AEs, and life-threatening AEs in the overall cohort was also similar to that in the ADVOCATE trial, though our study reported a higher rate of serious infections despite fewer infections overall.^5^ Patients with an initial eGFR < 15 ml/min had a higher rate of infections and serious infections compared to the overall cohort, possibly due to a higher rate of plasmapheresis in this group (8/15 patients; 53%). Further studies are needed to evaluate the safety of avacopan therapy in both subgroups. Regarding the 2 patients with leukopenia, an acute infectious cause was excluded. Neither patient received cyclophosphamide. Late-onset neutropenia is considered unlikely because neutropenia developed within the first 3 weeks of avacopan therapy and both patients had not previously received rituximab. Therefore, we suspect avacopan as a potential cause, though the data are associative rather than conclusive.

Importantly, unlike the rituximab induction arm of the ADVOCATE study, every patient in our study received maintenance treatment, predominantly using rituximab. The lack of a maintenance standard following rituximab induction was criticized within the ADVOCATE trial. Our data suggest that avacopan remains both safe and effective when combined with maintenance therapy, and its use in real-life setting does not result in foregoing maintenance treatment.

This study has limitations, including potential documentation gaps due to its retrospective data collection. The definition of relapse as either 1 major or 3 minor BVAS items, coupled with the intensification of immunosuppressive therapy, likely led to the lack of detection of minor disease activity. The retrospective nature and absence of structured visits or standardized schedules, as seen in randomized controlled trials, may have contributed to this oversight. Furthermore, not all included patients had a 6- or 12-month follow-up due to the predefined end of the retrospective analysis. Consequently, patients with relapses did not always have a complete follow-up and could not always meet the criteria for “remission” and “sustained remission” (Figure 1a), potentially inflating these rates. The study cohort was also heterogeneous, including patients treated with either rituximab or cyclophosphamide, both microscopic polyangiitis and granulomatosis with polyangiitis, and both relapsing and newly diagnosed AAV cases.

In summary, avacopan provides an effective and relatively safe therapy in our small real-world cohort, though vigilant monitoring for potential side effects is crucial. Additional research is required to determine the optimal treatment duration and understand the long-term effects of avacopan treatment. Finally, our data should spark prospective studies to evaluate avacopan in patients with an eGFR < 15 ml/min or those undergoing dialysis at diagnosis.

## Disclosure

AS and US received a study grant from CSL Vifor. AS, MB, US, US, VV, RK received honoraria for participation on Advisory Board for CSL Vifor. Additionally, this work was supported by grants SCHR 771/10-1 and SCHR 771/8-1 from the Deutsche Forschungsgemeinschaft to AS, grant 394046635 — SFB 1365 from the Deutsche Forschungsgemeinschaft to RK and AS, and ECRC grants to AS and RK.

## Supplementary Material

Supplementary File (PDF) **Supplementary Methods. Supplementary References. Figure S1.** Clinical and laboratory parameters at the time of diagnosis and during follow-up period. **Figure S2.** Course of estimated glomerular filtration rate (eGFR) in patients with an eGFR < 15 ml/min at the time of diagnosis during follow-up period. **Figure S3.** Clinical and laboratory parameters for each individual patient at the time of diagnosis and during follow-up period. **Figure S4.** Prednisolone-tapering for 12 months follow-up, whole population, relapses excluded. **Table S1.** Potential glucocorticoid-associated adverse events, defined terms according to the MedDRA (Medical Dictionary for Regulatory Activities) standards. **Table S2.** Safety results. Table S3. Individual decision to start avacopan therapy.

## Footnotes

Supplementary File (PDF)

**Supplementary Methods.**

**Supplementary References.**
**Figure S1.** Clinical and laboratory parameters at the time of diagnosis and during follow-up period. **Figure S2.** Course of estimated glomerular filtration rate (eGFR) in patients with an eGFR < 15 ml/min at the time of diagnosis during follow-up period. **Figure S3.** Clinical and laboratory parameters for each individual patient at the time of diagnosis and during follow-up period. **Figure S4.** Prednisolone-tapering for 12 months follow-up, whole population, relapses excluded. **Table S1.** Potential glucocorticoid-associated adverse events, defined terms according to the MedDRA (Medical Dictionary for Regulatory Activities) standards. **Table S2.** Safety results. **Table S3.** Individual decision to start avacopan therapy.
