# Supplementary material for: Avacopan in Anti-Neutrophil Cytoplasmic Autoantibodies–Associated Vasculitis in a Real-World Setting
Source: Kidney Int Rep. 2024 Jul 6;9(9):2803–8. doi: 10.1016/j.ekir.2024.07.007 (PMC11403094; doi:10.1016/j.ekir.2024.07.007)
Supplement: Supplementary File (PDF) — Supplementary Methods. Supplementary References. Figure S1. Clinical and laboratory parameters at the time of diagnosis and during follow-up period. Figure S2. Course of estimated glomerular filtration rate (eGFR) in patients with an eGFR < 15 ml/min at the time of diagnosis during follow-up period. Figure S3. Clinical and laboratory parameters for each individual patient at the time of diagnosis and during follow-up period. Figure S4. Prednisolone-tapering for 12 months follow-up, whole population, relapses excluded. Table S1. Potential glucocorticoid-associated adverse events, defined terms according to the MedDRA (Medical Dictionary for Regulatory Activities) standards. Table S2. Safety results. Table S3. Individual decision to start avacopan therapy. [file mmc1.pdf]

Supplementary Material

Supplementary Methods

Trial design and patient selection

This research is a multicenter observational study conducted in five major German vasculitis centers. Patients over 18 years of age were included with either relapsing or newly diagnosed granulomatosis with polyangiitis (GPA) or microscopic polyangiitis (MPA), treated with avacopan for a minimum of three months between February 2022 and June 2023 (intention to treat). They had to be tested positive for either myeloperoxidase (MPO) or proteinase-3 (PR3) antibodies, had at least one major Birmingham Vasculitis Activity Score (BVAS version 3) item or three minor BVAS items, had to be treated with rituximab or cyclophosphamide, but had no limitations concerning the estimated glomerular filtration rate (eGFR). For patients requiring mechanical ventilation, we pragmatically opened the capsules and administered the contents via the nasogastric tube. Due to the retrospective nature of the data, treatment selection and dosing was determined by the treating physician. The study was approved by the Institutional Review Board of the Charité Berlin (EA2/038/23).

Endpoints

The primary combined endpoint was remission, defined as a BVAS of 0 and ≤7.5mg prednisolone per day and no relapse at 6 months and sustained remission, defined as a BVAS of 0 at 6 and 12 months without relapse. Secondary endpoints included clinical and laboratory parameters such as hematuria (dipstick method), proteinuria/albuminuria, serum creatinine/eGFR, ANCA-titer, CRP, and relapse, defined as a return of vasculitis activity with at least one major BVAS item or three minor BVAS items AND intensification of immunosuppressive therapy. An increase in the prednisolone dose alone, in conjunction with clinical disease activity as measured by BVAS, was also defined as a relapse. Prednisolone dose adjustments in patients who did not clinically present as a relapse were not considered

as such. Other secondary endpoints included the cumulative total dose of glucocorticoids (both intravenous and oral prednisolone equivalent doses after a maximum of 52 weeks), and AEs, which were categorized as probably avacopan-associated (possibly leading to treatment discontinuation) and general AEs over the therapy period. Distinctions were made between serious AEs, life-threatening AEs, and glucocorticoid-associated AEs (defined terms according to the MedDRA (Medical Dictionary for Regulatory Activities) standards, Supplementary Table S1).[5] Treatment decisions regarding the prescription of avacopan for each patient were captured using a questionnaire completed by the treating physician. It was possible to select from six predefined reasons, from which all applicable options could be chosen.

Statistical analysis

The database was created using Microsoft Access, and statistical analyses and graphing were performed with the software 'R'. The study aimed to include the maximum number of available patients, which was still limited, thus forgoing formal sample size planning. All variables were descriptively analyzed for the entire cohort, using means with standard deviation, and absolute and relative frequencies for categorical data. The binary primary endpoint (remission, sustained remission) was presented as counts and percentages for the whole cohort. Adverse effects and the rationale for choosing avacopan therapy were reported in absolute and relative frequencies.

Supplementary Figures

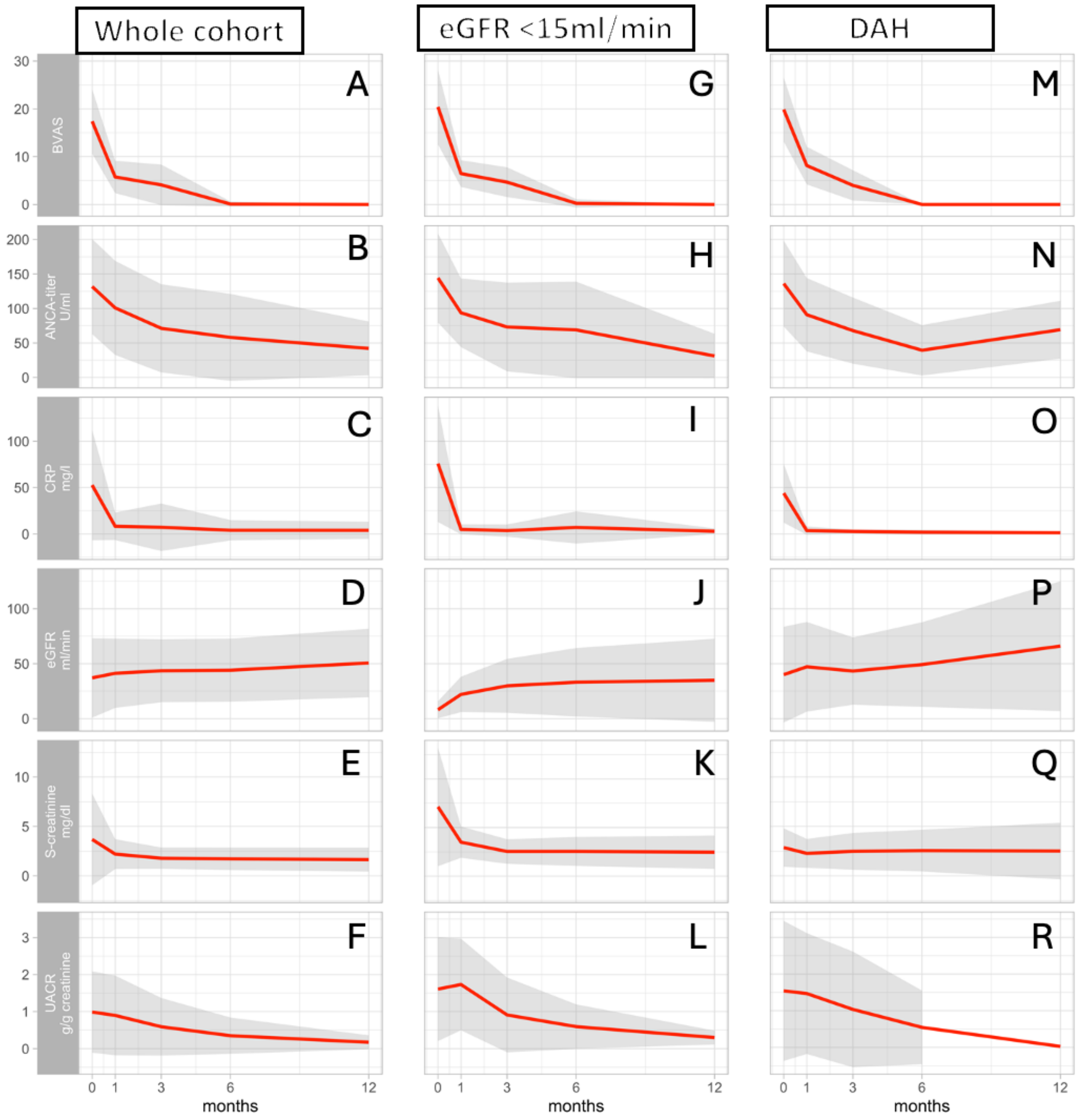

Supplementary Figure S1A-R: Clinical and laboratory parameters at the time of diagnosis and during follow-up period. Data are shown as mean ± SD. A-F: whole cohort; month 0 (N=39), month 1 (N=39), month 3 (N=39), month 6 (n=32), month 12 (n=23). G-L: patients with initial eGFR <15ml/min at the time of diagnosis; month 0 (n=15), month 1 (n=15), month 3 (n=15), month 6 (n=12), month 12 (n=9). M-R: patients with diffuse alveolar hemorrhage (DAH); month 0 (n=7), month 1 (n=7), month 3 (n=7), month 6 (n=6), month 12 (n=3). All clinical and laboratory parameters (A-I) improved over the follow-up period. Remarkably, a significant increase in the eGFR was observed in patients who presented with an eGFR <15ml/min at the time of diagnosis. Patients who required dialysis were considered to have an eGFR of 0ml/min (n=7). BVAS, Birmingham Vasculitis Activity Index; ANCA, anti-

neutrophil cytoplasmatic antibody; S-creatinine, Serum-creatinine; eGFR, estimated glomerular filtration rate; UACR, urine albumin to creatinine ratio; CRP, C-reactive protein.

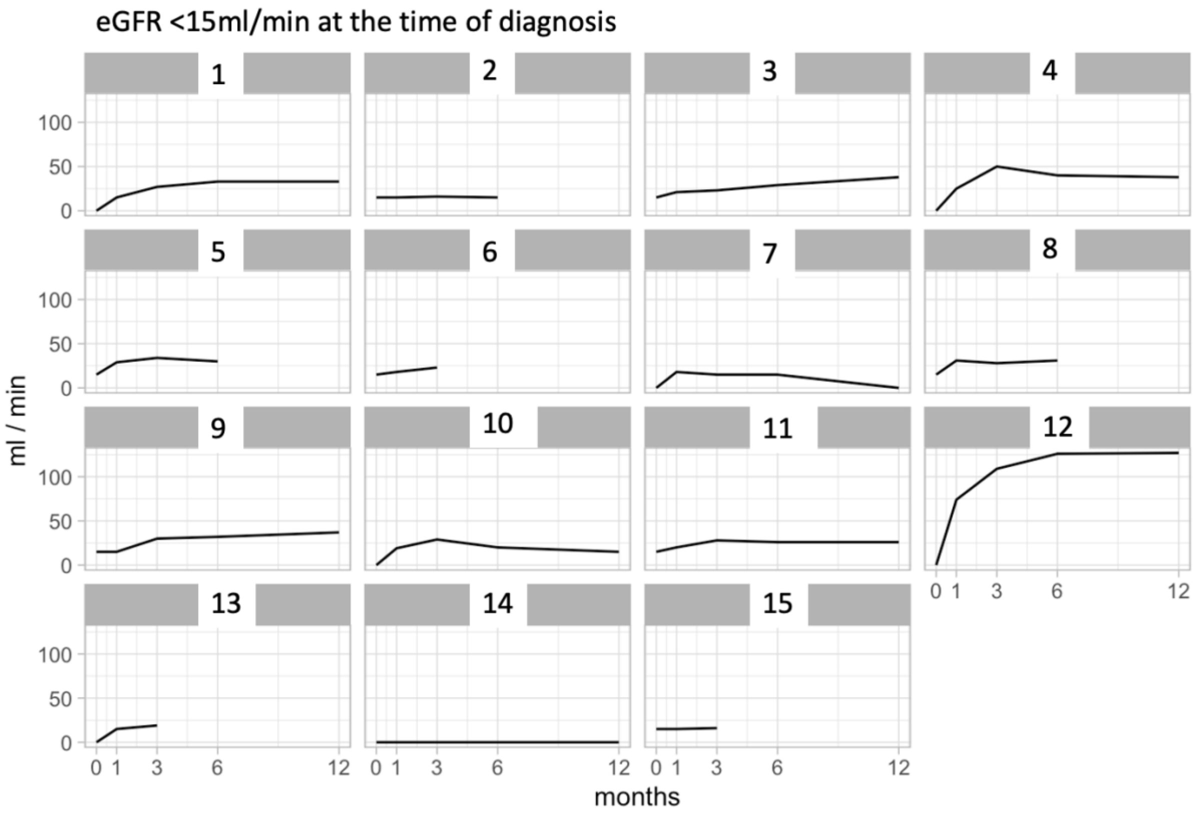

Supplementary Figure S2: Course of eGFR in patients with an eGFR <15ml/min at the time of diagnosis during follow-up period. Month 0 (n=15), month 1 (n=15), month 3 (n=15), month 6 (n=12), month 12 (n=9). eGFR, estimated glomerular filtration rate.

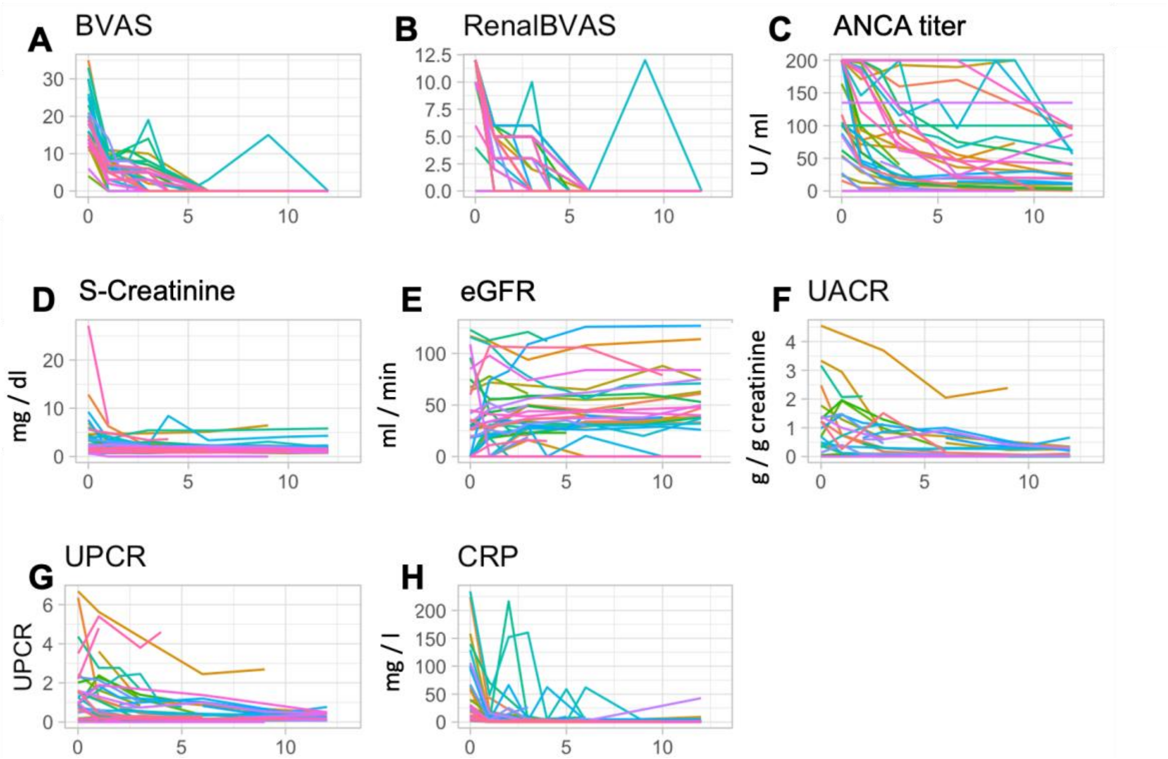

Supplementary Figure S3A-H: Clinical and laboratory parameters for each individual patient at the time of diagnosis and during follow-up period. Month 0 (N=39), month 1 (N=39), month 3 (N=39), month 6 (n=32), month 12 (n=23). BVAS, Birmingham Vasculitis Activity Index; ANCA, anti-neutrophil cytoplasmatic antibody; S-creatinine, Serum-creatinine; eGFR, estimatad glomerular filtration rate; UACR, urine albumin to creatinine ratio; UPCR, urine protein to creatinine ratio; CRP, C-reactive protein.

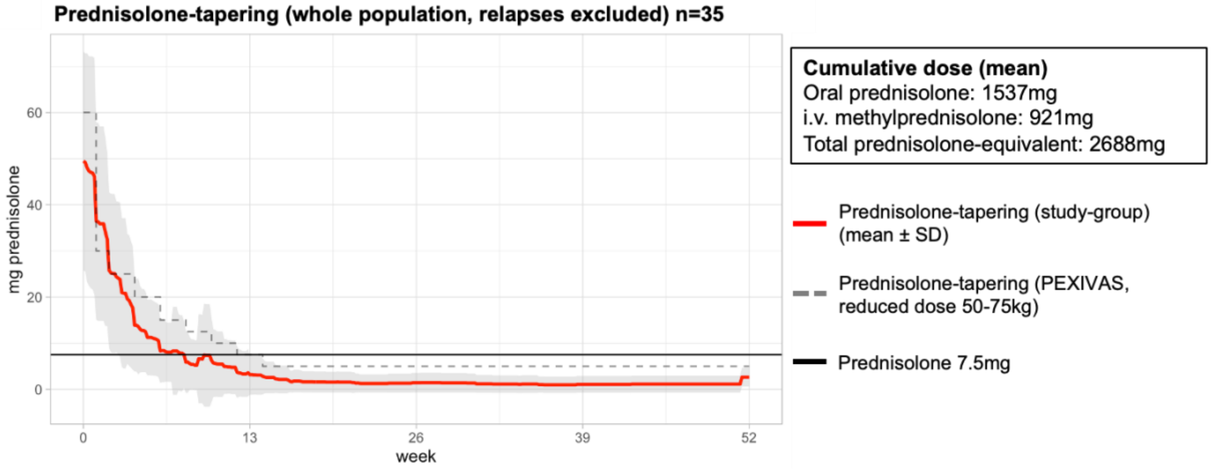

Supplementary Figure S4: Prednisolone-tapering during 12 months follow-up, whole population, relapses excluded. The average daily oral prednisolone dose (mean ± SD, represented by the red solid line) is shown for the entire study population excluding patients with relapses (n=35). For comparison, the prednisolone tapering regimen from the PEXIVAS study (reduced dose for 50-75kg of body weight, mean, depicted by the dashed line) is illustrated. The black line indicates a prednisolone dose of ≤7.5mg. Additionally, the oral prednisolone dose, intravenous (i.v.) methylprednisolone dose, and the total prednisolone equivalent dose are provided.

Supplementary Tables

Supplementary Table S1: Potential Glucocorticoid-associated AEs, defined terms according to the MedDRA (Medical Dictionary for Regulatory Activities) standards.

**Organ system**	**MedDRA preferred terms**
Cardiovascular	Peripheral oedema, peripheral swelling, oedema, hypertension, blood pressure increased, arteriosclerosis, hypertensive emergency, hyperlipidaemia, hypercholesterolaemia, dyslipidaemia, hypertriglyceridaemia, blood cholesterol increased, low density lipoprotein increased hypervolaemia, fluid retention, angina pectoris, cardiac failure, acute myocardial infarction, cardiovascular insufficiency, congestive cardiomyopathy, myocardial ischemia, myocardial infarction
Infections	All serious infections
Gastrointestinal	Acute pancreatitis, duodenal ulcer, erosive gastritis, gastrointestinal disorder
Psychological	Insomnia, depression, anxiety, depressed mood, mania, affective disorder, agitation, libido decreased, nervousness, confusional state, irritability, major depression, mental status changes, mood altered, poor quality sleep
Endocrine/metabolic	Hyperglycaemia, hypokalaemia, diabetes mellitus, type 2 diabetes mellitus, central obesity, diabetes mellitus inadequate control, glucose tolerance impaired, waste circumference increased, weight increased, blood glucose increased, Cushingoid, adrenal insufficiency, Cushing's syndrome, menorrhagia, metrorrhagia, gynaecomastia, influenza like illness, systemic inflammatory response syndrome
Dermatological	Dermatitis acneiform, acne, ecchymosis, hirsutism, skin atrophy, skin striae, increased tendency to bruise
Musculoskeletal	Myopathy, osteopenia, osteoporosis, osteonecrosis, muscle atrophy, muscular weakness, hip fracture, humerus fracture, lower limb fracture, tendon rupture, wrist fracture, lumbar vertebral fracture, spinal compression fracture
Ophthalmological	Cataract, hypertensive retinopathy, cataract, glaucoma, open angle glaucoma, intraocular pressure increased

Supplementary Table S2: Safety results.

Events	Overall cohort N = 39 – No. (%)	eGFR <15ml/min N = 15 – No. (%)	DAH N = 7 – No. (%)
Any adverse event	34 (87)	13 (87)	6 (86)
Any serious adverse event*	14 (36)	6 (40)	2 (28)
Life-threatening adverse event⁺	3 (8)	1 (7)	1 (14)
Death#	1 (3)	0 (0)	0 (0)
Any infection	14 (36)	9 (60)	3 (43)
Any serious infection§	6 (15)	4 (27)	1 (14)
Any potential glucocorticoid-associated adverse event&	24 (62)	11 (73)	4 (57)
Cardiovascular	15 (38)	6 (40)	2 (29)
Infectious	10 (26)	7 (47)	2 (29)
Gastrointestinal	4 (10)	3 (20)	0 (0)
Psychological	1 (3)	0 (0)	0 (0)
Endocrine/metabolic	10 (26)	4 (27)	1 (14)
Dermatological	1 (3)	1 (7)	0 (0)
Musculoskeletal	0 (0)	0 (0)	0 (0)
Ophthalmological	0 (0)	0 (0)	0 (0)
Discontinuation of avacopan therapy (total)	9 (23)	4 (27)	2 (29)
Discontinuation of avacopan therapy due to adverse event	8 (21)	3 (20)	2 (29)

*Serious adverse events were categorized as those leading to death, posing immediate life-threatening risks, necessitating or extending hospitalization, causing lasting or clinically meaningful disability or incapacity, involving birth defects, or comprising significant occurrences that require intervention/treatment to prevent the aforementioned outcomes.

⁺ Life-threatening adverse events were considered as such when the attending physician determined that the patient was at substantial risk of dying at the time of the adverse event. These included a tonic-clonic seizure, an enterococcal sepsis during a period of neutropenia with respiratory insufficiency, intermittent hemodialysis requirement, and cardiac decompensation, as well as a (peri)myocarditis with severely reduced left ventricular ejection fraction during a cardiac relapse of AAV and iatrogenic pericardial tamponade with pigtail catheter placement following coronary angiography.

# One patient died of unknown causes, a situation only reported externally, after the patient ceased to attend the cyclophosphamide therapy.

§ This definition aligns with that of serious adverse events, with a specific focus on infections. These include sinusitis, bloodstream infection, enterococcal sepsis, soft tissue infection, rotavirus enteritis, and pneumonia.

& Defined terms according to the MedDRA (Medical Dictionary for Regulatory Activities) standards (Supplementary Table S2)[5]. One patient could have multiple events.

eGFR, estimated glomerular filtration rate; DAH, diffuse alveolar hemorrhage.

Supplementary Table S3: Individual decision to start avacopan therapy.

Therapy decision*	N = 39 – No. (%)
Improving therapeutic response / overall outcome	21 (54)
Improving renal response / outcome	23 (59)
Diabetes mellitus or adipositas	11 (28)
Reduction in the cumulative dose of glucocorticoids	30 (77)
Contraindications for glucocorticoids	1 (3)
Intensification of therapy for uncontrolled disease / desire for higher immunosuppressive efficacy / relapse	20 (51)

*Treatment decisions for each patient were captured using a questionnaire completed by the treating physician. Multiple selections were allowed.
